# Extension of *Drosophila* Lifespan by *Rhodiola rosea* through a Mechanism Independent from Dietary Restriction

**DOI:** 10.1371/journal.pone.0063886

**Published:** 2013-05-21

**Authors:** Samuel E. Schriner, Kevin Lee, Stephanie Truong, Kathyrn T. Salvadora, Steven Maler, Alexander Nam, Thomas Lee, Mahtab Jafari

**Affiliations:** Department of Pharmaceutical Sciences, University of California Irvine, Irvine, California, United States of America; Leibniz Institute for Age Research - Fritz Lipmann Institute (FLI), Germany

## Abstract

*Rhodiola rosea* has been extensively used to improve physical and mental performance and to protect against stress. We, and others, have reported that *R. rosea* can extend lifespan in flies, worms, and yeast. However, its molecular mechanism is currently unknown. Here, we tested whether *R. rosea* might act through a pathway related to dietary restriction (DR) that can extend lifespan in a range of model organisms. While the mechanism of DR itself is also unknown, three molecular pathways have been associated with it: the silent information regulator 2 (SIR2) proteins, insulin and insulin-like growth factor signaling (IIS), and the target of rapamycin (TOR). In flies, DR is implemented through a reduction in dietary yeast content. We found that *R. rosea* extract extended lifespan in both sexes independent of the yeast content in the diet. We also found that the extract extended lifespan when the SIR2, IIS, or TOR pathways were genetically perturbed. Upon examination of water and fat content, we found that *R. rosea* decreased water content and elevated fat content in both sexes, but did not sensitize flies to desiccation or protect them against starvation. There were some sex-specific differences in response to *R. rosea*. In female flies, the expression levels of glycolytic genes and *dSir2* were down-regulated, and NADH levels were decreased. In males however, *R. rosea* provided no protection against heat stress and had no effect on the major heat shock protein HSP70 and actually down-regulated the mitochondrial HSP22. Our findings largely rule out an elevated general resistance to stress and DR-related pathways as mechanistic candidates. The latter conclusion is especially relevant given the limited potential for DR to improve human health and lifespan, and presents *R. rosea* as a potential viable candidate to treat aging and age-related diseases in humans.

## Introduction

The root extract of *Rhodiola rosea*, also known as the golden root, has been widely used in traditional and integrative medical practices in Europe and Asia, where it has been purported to mediate a variety of beneficial effects in humans, such as improved mood, improved physical and mental stamina, and enhanced protection against high altitude sickness [1]. The extract has also been reported to protect against tumor progression in mice, improve endurance in rats, improve blood glucose profiles in diabetic mice, and protect snail eggs against oxidative stress, heat, and heavy metals [2]–[5]. Our group has previously reported that *R. rosea* can extend the lifespan of the fruit fly, *Drosophila melanogaster*, protect flies and human cultured cells against oxidative stress, and decrease the production of reactive oxygen species in isolated fly mitochondria [6]–[8]. In addition to the fly, the extract has also been shown to extend lifespan in the worm, *Caenorhabditis elegans* [9], and in the yeast, *Saccharomyces cerevisiae* [10]. These observations demonstrate that *R. rosea* lifespan-extending properties are not limited to the fly, and suggest that it may be a viable treatment to slow aging and abrogate age-related diseases in a range of species, potentially including humans.

The molecular action of *R. rosea* is not known, though its effects in worms suggest that it may act through hormesis [9], where pretreatment of a mildly toxic compound induces defense systems that further protect the organism against any additional stress [11]. Contrary to this, we have shown that *R. rosea* is able to confer protection against oxidative stress in cultured cells at doses far below what is required to activate antioxidant defenses [8]. We then proposed an alternate hypothesis, that *R. rosea* may act through a pathway related to dietary restriction (DR), e.g., as a DR mimetic. To date, DR, defined as a decreased total caloric intake in the absence of malnutrition, is considered the most robust non-genetic treatment for improving health and extending lifespan in model organisms. This treatment has been shown to benefit nearly all organisms tested, from yeast to primates [12]–[15], though a recent study has questioned its effectiveness in primates [16]. Like *R. rosea*, the molecular mechanism of DR is not known, however, three different but overlapping molecular pathways, sometimes termed nutrient-sensing pathways, are thought to be involved in the mechanism of DR. These molecular pathways are the silent information regular 2 (SIR2) proteins [17], the target of rapamycin (TOR) [18], and insulin and insulin-like growth factor signaling [19]. Given the robustness of DR in model systems, there has been a significant effort to identify compounds that could mimic the action of DR at the molecular level. Three prominent suggested candidates are resveratrol, rapamyacin, and metformin [20]. Of these three, metformin, a highly prescribed medication for type 2 diabetes, appears to be the most promising. While metformin appears to mimic the molecular effects of DR in mice [21], it has had mixed results in invertebrate models; it extended mean lifespan in worms [22], but had no positive effect in flies [23]. Nevertheless, a plausible mode of action of *R. rosea* could be that it acts as a DR mimetic.

The purpose of this work was to examine such a possibility. We found that *R. rosea* extended lifespan independent of dietary yeast content in flies, the method by which DR is imposed in flies. The extract also extended lifespan when any of the 3 nutrient-sensing pathways were perturbed, demonstrating that it acts independently from these pathways as well. *Rhodiola rosea* exhibited no effect in male flies in 4 DR-related parameters examined: glycolysis, *dSir2* expression, NAD^+^/NADH ratios, and total soluble protein levels. Though, in females the extract down-regulated glycolytic enzymes and elevated NAD^+^/NADH ratio, suggesting the possibility of a partial DR effect in females. Nonetheless, *R. rosea* was able to extend lifespan in flies while exhibiting no experimental outcome consistent with DR, refuting our original hypothesis, and demonstrating that *R. rosea* acts through a mechanism unrelated to DR.

## Results

### Extension of Lifespan Independent of Dietary Yeast Content

**Figure 1:**
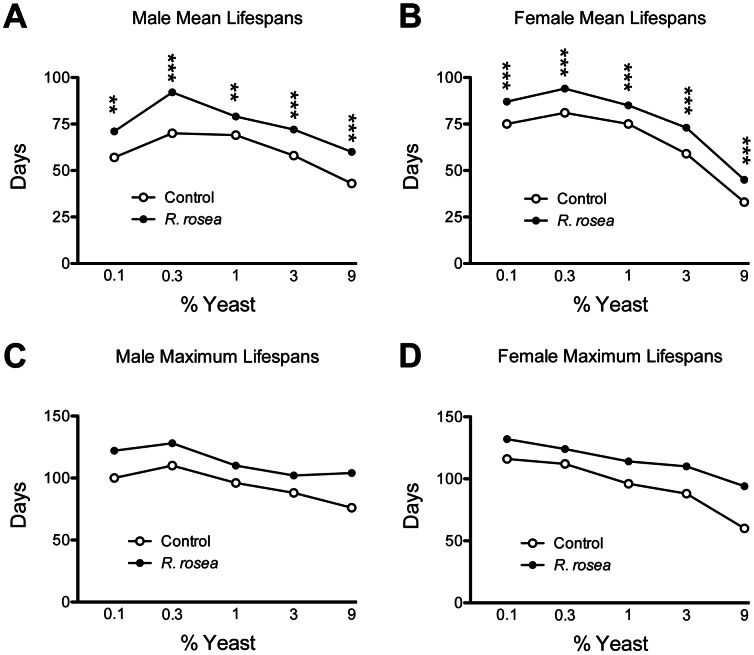
Extension of lifespan by *Rhodiola rosea* independent of dietary yeast content. A and B. *R. rosea* feeding increased both mean lifespan and **C** and **D**, maximum lifespans in both sexes. The magnitude of mean lifespan increase for each dietary group are as follows: Males: 0.1%: 25%; 0.3%: 31%; 1%: 14%; 3%: 24%; 9%: 40%; Females: 0.1%: 16%; 0.3%: 16%; 1%: 13%; 3%: 24%; 9%: 36%. **P<0.001, ***P<0.0001, Mann-Whitney nonparametric test. Sample sizes for the control groups and treated groups respectively were as follows: Males: 0.1%: 120, 105; 0.3%: 112, 111; 1%: 110, 114; 3%: 116, 100; 9%: 99, 108; Females: 0.1%: 103, 121; 0.3%: 104, 112; 1%: 112, 105; 3%: 107, 122; 9%: 117, 121.

The principal objective of this work was to examine whether *R. rosea* extended lifespan by mimicking the action of DR. In flies, DR is typically implemented by decreasing the percentage of yeast in the diet [18], [24]. Lifespan increases as dietary yeast is decreased up to a point where yeast content becomes so low that malnutrition compromises lifespan. We varied the yeast content by a factor of 3, from 0.1% up to 9%. *Rhodiola rosea* extended lifespan in both sexes at all dietary yeast contents examined (Figure 1).

### Extension of Lifespan with Perturbed Nutrient Sensing

**Figure 2:**
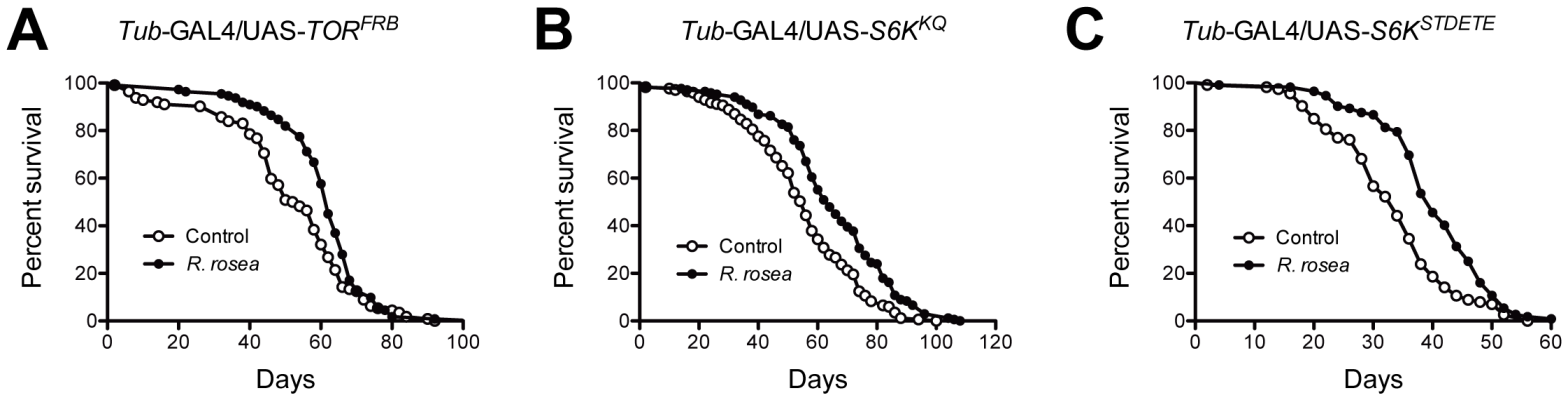
Extension of lifespan by *Rhodiola rosea* when the TOR pathway is perturbed. A. *R. rosea* extended lifespan when TOR was inhibited, *P* = 0.003, n = 111 controls, 112 treated; **B**. the downstream S6 kinase was inhibited, *P*<0.0001, n = 167 controls, 169 treated; and **C**. when S6 kinase was constitutively activated, *P*<0.0001, n = 113 controls, 112 treated. The increases in mean lifespan due to *R. rosea* feeding were 17%, 19%, and 22%, respectively. *P*-values were calculated with the Mantel-Cox Log-Rank test.

**Figure 3:**
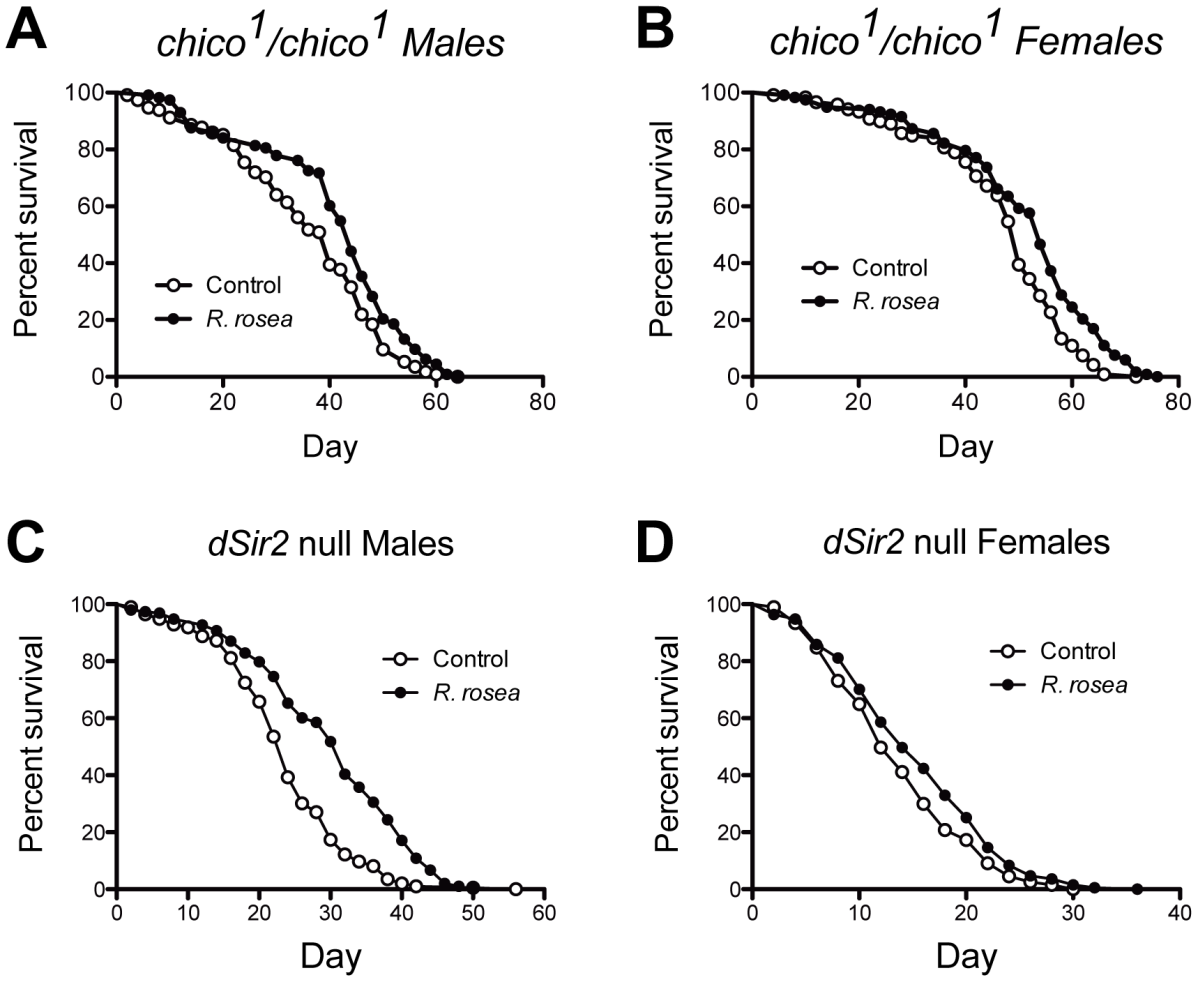
*Rhodiola rosea* extended lifespan when the SIR2 and IIS pathways are blocked. *Rhodiola rosea* extended lifespan in the absence the insulin receptor substrate in both **A**, males, *P* = 0.004, and **B**, females, *P* = 0.0004. The extract also extended lifespan in the absence of the principal *Drosophila* Sir2 protein, dSIR2, in both **C**, males, *P*<0.0001 and **D**, females, *P*<0.05. *P*-values were calculated with the Mantel-Cox Log-Rank test. Increases in mean lifespan due to *R. rosea* feeding were as follows: *chico^1^* males: 14%; *chico^1^* females: 9%; *dSir2* males: 27%, *dSir2* females: 11%. Sample sizes for the control groups and treated groups respectively were as follows: *chico^1^* males: 113, 114; *chico^1^* females: 119, 118; *dSir2* males: 196, 193, *dSir2* females: 197, 191.

The actual manner in which DR extends lifespan is not known. However, 3 molecular pathways have been associated with its action: silent information regular 2 (SIR2) proteins [17], the target of rapamycin (TOR) [18], and insulin and insulin-like growth factor signaling [19]. The SIR2 proteins are a group of NAD^+^-dependent deacetylases that regulate gene expression in response to cellular nutrient levels [25], . The Target of Rapamycin is a serine/threonine protein kinase that is part of a complex that senses nutrient levels and growth factors and then regulates many cellular functions, such as cell growth, proliferation, and protein synthesis. Insulin and insulin-like growth factor signaling responds to circulating signaling molecules and regulates the synthesis of proteins, fats, and glycogen [27]. We examined the requirement of each of these pathways in the ability of *R. rosea* to extend lifespan by using flies in which the pathways were genetically perturbed. These were flies in which the TOR pathway was both inhibited and constitutively activated (Figure 2), deficient in the insulin receptor substrate, *chico* (Figure 3A and B), and deficient in the major *Drosophila* Sir2 homolog, dSir2 (Figure 3C and D). In all cases, *R. rosea* was able to extend lifespan in both male and female flies.

### Sex Specific Effects on NAD^+^/NADH Ratios, Glycolytic Genes, and *dSir2* Expression

**Figure 4:**
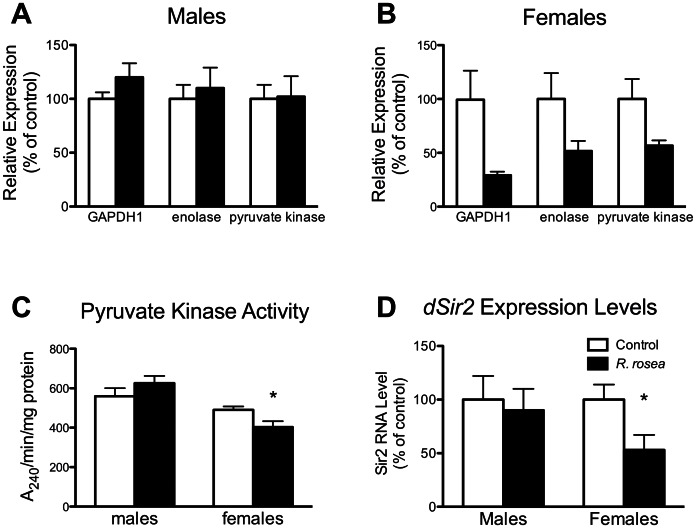
The effect of *Rhodiola rosea* on glycolytic and dSir2 gene expression in *w^1118^* flies. A. The expression levels of 3 glycolytic genes, GAPDH1, enolase, and pyruvate kinase, were unaffected in males. *P*>0.05 for diet, two-way ANOVA. **B.** However, these 3 genes were all down-regulated in females. *P*<0.001 for diet, two-way ANOVA. **C.** Pyruvate kinase enzyme activity is down regulated, approximately 20%, in females, but not males, consistent with our gene expression data. *P<0.05, Students t test. **D.** Expression levels of *dSir2* were unaffected in males, but down-regulated in females. *P<0.05, Students t test. Data are mean ± sem, n = 6 groups of 25 flies.

We then examined several physiological markers associated with DR, such as decreased NADH [25], decreased glycolytic gene expression [28], [29], and elevated *dSir2* expression [30]. In males, *R. rosea* had no effect on any of these parameters (Figures 4A, C, and D, and 5A, B, and C). In females, *R. rosea* decreased NADH levels resulting in elevated NAD^+^/NADH ratios, decreased glycolytic gene expression, but surprisingly decreased *dSir2* expression (Figures 4B, C, and D, and 5B and C).

**Figure 5:**
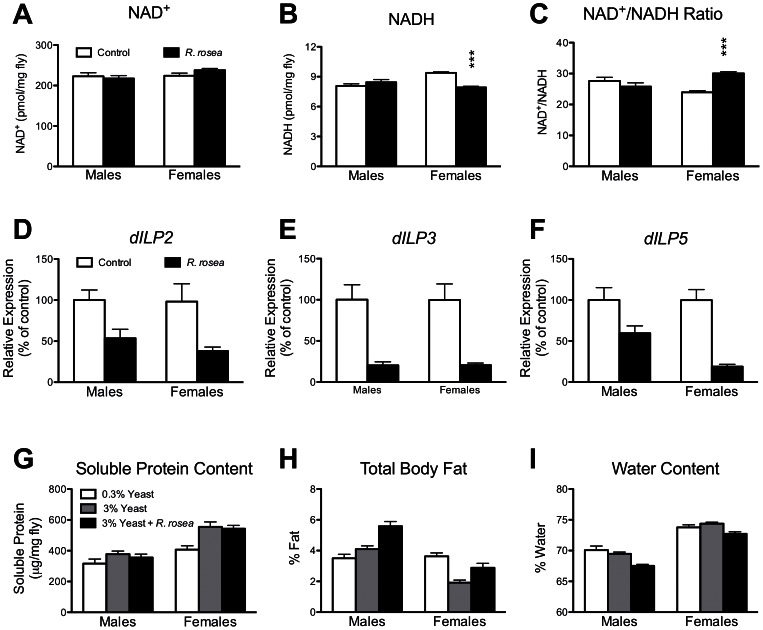
The effect of *Rhodiola rosea* on NAD^+^ and NADH levels, *dilp* expression, and protein, fat, and water content. A. Total NAD^+^ content was unaffected by *R. rosea* feeding, however, **B.** NADH levels were significantly decreased in females, but not males given *R. rosea*. **C.** The ratio of NAD^+^ and NADH, calculated from the values in panels A and B, are significantly elevated in females, but not males. Data are mean ± sem, n = 6. ***P<0.0001 for interaction between sex and diet, two-way ANOVA, *P*<0.001 between control and *R. rosea*-fed females, Bonferroni posttest. *Rhodiola rosea* feeding resulted in down-regulation of **D.** *dilp2*, *P* = 0.001, **E.** *dilp3*, *P*<0.0001 and **F.** *dilp5 P*<0.0001, in both sexes. *P*-values are for diet, 2-way ANOVA, n = 6 groups of 25 flies for each bar. **G.** Soluble protein was decreased in females due to DR, but was unaffected by *R. rosea* feeding, *P*<0.001. **H.** Fat content was unaffected by DR in males, but was elevated by DR in females, *P*<0.001. *Rhodiola rosea* elevated fat content in both sexes, *P*<0.001 for males, *P*<0.05 for females. **I.** Water content was unaffected by DR, but was decreased by *R. rosea* in both sexes, *P*<0.01 for males and *P*<0.05 for females. *P*-values were calculated by Bonferroni posttests, 2-way ANOVA relative to the 3% yeast control group. For all experiments, n = 6 groups of 10–50 flies per bar.

### Down-regulation of Ageing-related *Drosophila* Insulin-like Peptides

We further examined the role that IIS might play in the action of *R. rosea* by examining the expression levels of the *Drosophila* insulin-like peptides (dILPs). *Drosophila* has 7 dILPs, and the expression levels of 3 of them, dILPs 2, 3, and 5, have been inversely related to lifespan extension in flies [31], [32]. However, only the down-regulation of dILP5 has been seen in DR treated flies [33]. In the case of *R. rosea* feeding, all 3 were down-regulated in both sexes (Figure 5D, E, and F).

### Sex Specific Effects on Soluble Protein, Water, and Fat Content

Dietary restriction in flies has been shown to result in decreased levels of soluble proteins [34]. We observed the same result in DR-treated females (0.3% dietary yeast) when compared to controls (3% dietary yeast). However, no such change was observed in either sex in flies fed *R. rosea* (Figure 5G) relative to controls (both diets at 3% yeast). We then examined the effect of *R. rosea* on fat and water content. Dietary restricted males exhibited no change in fat content relative to control diet-fed animals, though DR-treated females exhibited a marked increase in fat content (Figure 5H). In both sexes, *R. rosea* elevated fat content (Figure 5H). We then examined whether the elevated fat was displacing water in the flies, and found this to be the case in both sexes (Figure 5I). Water content was unchanged in DR-treated flies relative to control diet-fed flies (Figure 5I).

### The Absence of Effect on Desiccation, Starvation and Heat Tolerance

**Figure 6:**
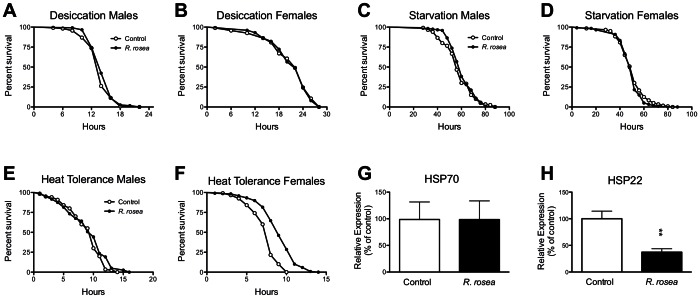
The effect of *Rhodiola rosea* on the tolerance towards starvation, desiccation, and heat. A 2-week feeding of *R. rosea* had no ability to protect males or female flies against **A, B,** desiccation or **C, D,** starvation. *P*>0.05 for all groups. Mantel-Cox Log-Rank test. Sample sizes for the control groups and treated groups respectively were as follows: desiccation males: 114, 118; desiccation females: 94, 101; starvation males: 120, 118; starvation females: 120, 120. **E.** *Rhodiola rosea* did not protect males against exposure to 37°C, but did so in **F.** females, *P*<0.0001, Mantel-Cox Log-Rank test. Sample sizes for the control groups and treated groups respectively were as follows: males: 120, 120; females: 120, 119. **G.** *Rhodiola rosea* had had no effect of HSP70 expression but **H.** down-regulated HSP22 in males, *P* = 0.004, Mann-Whitney nonparametric test, n = 5 groups of 25 flies for the controls and 6 groups of 25 flies for the treated flies.

Given our results that *R. rosea* decreased water content and elevated fat content, we investigated whether the extract would sensitize flies to desiccation and/or protect against starvation. *Rhodiola rosea* had no effect on either parameter (Figure 6A, B, C, and D). This was somewhat surprising, since *R. rosea* has been shown to protect against many different types of stresses in other organisms [2]–[5], and against oxidative stress in flies [7]. Given these results, we decided to determine whether *R. rosea* could protect against heat. The extract did so in females, but not in males (Figure 6E and F). This again was surprising since *R. rosea* extended lifespan in both sexes. We then investigated the effect of *R. rosea* on the expression levels of the major heat shock protein HSP70 and the mitochondrial HSP22 in males. We found no effect of *R. rosea* on HSP70 expression and a decrease in expression of HSP22 (Figure 6G and H).

## Discussion

Previously, we had reported that the root extract of *Rhodiola rosea* could extend the lifespan of the fruit fly, *Drosophila melanogaster* [6], [7]. Our findings are supported by similar results in the worm, *Caenorhabditis elegans* [9], and in the yeast, *Saccharomyces cerevisiae* [10]. However, the molecular mechanism by which *R. rosea* extends lifespan is not known. Here, we examined whether *R. rosea* acts through molecular pathways associated with dietary restriction (DR), a reduction in caloric intake without malnutrition, which is considered to be the most robust mechanism for extending lifespan and improving health in model organisms [12]–[15]. Our results show that *R. rosea* acts in a manner unrelated to DR.

Dietary restriction is imposed in mammals by decreasing the amount of food provided [35]. However, in flies it is typically undertaken by decreasing the percentage of yeast in the diet [24]. Decreasing the dietary yeast content then increases lifespan up to a point where a further reduction begins to shorten lifespan (Figure 1). This latter shortening of lifespan is likely due to an excessive restriction of protein and/or sub-optimal intake of other nutrients. If a compound or extract acts in a DR-dependent manner, we would expect a maximal effect on lifespan at the highest dietary yeast content, and then a diminished effect as decreased dietary yeast concentrations increased lifespan (Figure 1, controls). We would also expect that a DR effect would further compromise lifespan at the lowest dietary yeast content, as the animals are in a nutritionally deprived state. Such a DR-dependent mode of action has been clearly demonstrated for the target of rapamycin (TOR) [18]. However, in our work, *R. rosea* extended lifespan at all dietary yeast contents, even when low dietary yeast content shortens lifespan (Figure 1). This is contrary to our predictions of a DR-related effect. Furthermore, while the physiological effects of DR have been extensively documented, its molecular mechanism, like *R. rosea*, is not known. In addition to TOR mentioned above, two other molecular pathways have been implicated in action of DR: the silent information regulator 2 proteins (SIR2), and insulin and insulin-like growth factor signaling (IIS) [19], [26]. The ability of *R. rosea* to still extend lifespan in flies in which these pathways were perturbed (SIR2 and IIS were blocked, while TOR signaling was both blocked and activated) strongly supports a DR-independent mode of action (Figures 3 and 4).

To further explore a potential DR effect by *R. rosea*, we examined four other parameters associated with DR: down-regulation of glycolysis, up-regulation of *dSir2*, elevated NAD^+^/NADH ratios, and decreased soluble protein levels [25], [28]–[30], [34]. In male flies, *R. rosea* had no effect on any of these parameters (Figures 4A, C, and D, 5A, B, C, and G). Thus, in males, the examination of 8 different parameters (these 4 parameters plus dietary yeast content, and the 3 nutrient sensing pathways) showed no *R. rosea* induced DR effect. In females, we did see some DR-related effects: down-regulation of glycolytic genes (Figure 4B and C), and elevated NAD^+^/NADH ratios (Figure 5C). However *R. rosea* did not decrease soluble protein levels in females (Figure 5G), and actually decreased *dSir2* expression (Figure 4D), both effects inconsistent with DR. Despite the mixed results in females, the ability of *R. rosea* to extend lifespan in males without *any* associated DR effects, demonstrates that *R. rosea* is fully capable of extending lifespan in a DR-independent manner.

The feeding of *R. rosea* to flies did lead to some surprising results such as the decrease in water content (Figure 5I). Dehydration is thought to be a significant contributor to fly death, and selection for postponed aging has been found to result in an increased tolerance to desiccation [36]. Since *R. rosea* increases lifespan, we expected that if it had any effect on water content it would be an increase. This decreased water content may have resulted from displacement of water by the elevated fat seen in *R. rosea*-fed flies (Figure 5H). The altered water and fat contents, the lack of any effect on desiccation and starvation tolerance, and the inability to protect against heat were surprising observations given the evidence that *R. rosea* protects against stress in a variety of conditions [4], [5], [7], [8]. These types of results are not unique to *R. rosea.* For instance, the finding that mitochondrial HSP22 was down-regulated is similar to what we observed with another botanical, *Rosa damascena*, which also extended fly lifespan [37]. While we have previously shown that *R. rosea* protected against oxidative stress in flies and cultured cells [7], [8], the role of oxidative stress in aging has been somewhat marginalized recently [38], [39]. In flies, it has been argued that elevated oxidative stress resistance is valuable only in shorter-lived strains, and loses its beneficial effects in longer-lived, presumably healthier flies [40]. Given our results that *R. rosea* extends lifespan in very long-lived DR-treated flies (Figures 1 and 2), we could argue that even the protection against oxidative stress imparted by the extract may be unimportant in its ability to extend lifespan.

An interesting and important observation is the down-regulation of *dilp2*, *3*, and *5* (Figure 5D, E, and F). We argue that *R. rosea* does not act through IIS, due to its ability to extend lifespan when the pathway is blocked by the absence of the insulin receptor substrate, *chico* (Figure 3A and B). Nevertheless, the down-regulation of these proteins may be instrumental in its action, as their expression levels, particularity those of *dilp2*, are inversely related to lifespan in fruit flies. For example, over-expression of *dilp6* in the fat body extended fly lifespan and decreased the expression levels of both *dilp2* and *5* [41], while over-expression of *dFOXO,* which also extends lifespan, decreased the expression levels of *dilp2* [31]. Over-expression of uncoupling protein-3 elevated DILP2 protein levels and shortened lifespan [32]. The most direct test of the role of these proteins in aging was the deletion of the neurosecretory cells that produce *dilp2*, *3*, and *5*, which resulted in an extended lifespan [42]. However, the phenotypes observed in this experiment only partially overlap those seen in flies fed *R. rosea*. Flies that lacked *dilp2*, *3*, and *5* expression had an elevated fat content, were sensitized to heat, and exhibited enhanced protection against oxidative stress and starvation [42]. In flies fed *R. rosea*, we did see protection against oxidative stress [7] and an elevated fat content (Figure 5H), but we saw no protection against starvation and no sensitivity to heat (Figure 6E and F). Actually, in our case, females had an enhanced tolerance to heat (Figure 6F. Therefore, it isn’t clear exactly what the role of decreased *dilp* expression is in the action of *R. rosea*. A possible explanation for the differences in outcomes between Broughton’s experiment and ours is the degree in which the *dilp2*, *3*, and *5* are down-regulated. In their case, the expression of these genes are completely absent, while in ours their expression levels are decreased to 25–50% of baseline. Alternatively, *R. rosea* may act on other targets, which might mitigate the effects of decreased *dilp* expression. Future experiments in flies with elevated or decreased *dilp* expression levels will help inform us on the role of the *dilp2*, *3*, and *5* in the action of *R. rosea*.

The root extract of *R. rosea* used in this study is composed of at least 140 compounds [43]. The active compounds in the extract are not known for certain, though they are thought to include salidroside, tyrosol and 3 rosavin compounds [1]. The extract also has a significant polyphenol content that may contribute to its activity [43]. While we are currently searching for the extract’s active compound with respect to lifespan extension, it may be that there is no single compound responsible, and such a search may be somewhat misguided due to the complexity of aging. There are likely thousands of genes that play role in the lifespan of an organism [44]. Therefore, it may not be particularly successful to search for single anti-aging compounds, as most compounds would have a limited number of molecular targets. One exception would be compounds that mimic DR, as such molecules could modulate the expression of many downstream targets. However, the recent finding that DR may not extend lifespan in primates [16] may lower the enthusiasm regarding DR mimetic compounds. The use of complex natural products to screen for lifespan-extending effects then presents an advantage, as these extracts contain many molecules that could simultaneously target multiple molecular pathways.

Our initial hypothesis was that *R. rosea* extended fly lifespan by acting as a DR mimetic by targeting one of 3 DR-related pathways. Here, we tested this hypothesis through a series of genetic, dietary, and phenotypic analyses. As a result of our findings, we argue that *R. rosea* extends fly lifespan through a mechanism independent from DR, though that mechanism has not yet been identified. We also show that *R. rosea* extended lifespan in both sexes. Many aging studies in *Drosophila* are conducted only with males, and often when both sexes are used, sex-specific differences are observed. For example, the complete loss of *chico* extended lifespan only in females [45], whereas *Rosa damascena* and curcumin both showed sex-specific effects on lifespan depending on the strain used [37], [46]. The ability of *R. rosea* to extend lifespan in DR-treated flies shows that the extract acts in long-lived, presumably very healthy flies, and doesn’t simply make sick flies healthier. Of particular note is our finding that *R. rosea* feeding when combined with a low dietary yeast content (0.3%) resulted in some of the longest lived laboratory flies, with mean lifespans of over 90 days and maximum lifespans exceeding 120 days in both sexes (Figure 1 and Table S1). Thus, the extract has the potential to improve longevity in humans of both genders who are healthy and non-obese. The additive activity of DR and *R. rosea* also shows that the extract could be used in conjunction with known DR-mimetics, or other compounds which target DR-related pathways, to provide even further benefits in obese or sick individuals. Therefore, *R. rosea* may present a viable and potentially powerful therapy for aging and age-related diseases in a broad group of patients. Our future work will be to identify the precise molecular target of the extract using other genetic models in combination with microarray and proteomic techniques, identify its active compounds, and study its interaction with other known lifespan extending treatments.

## Materials and Methods

### Fly Strains

The *w^1118^* control and *w^1118^*, *Sir2^2A-7-11^*; *chico^1^*; and *Tub*-GAL4 flies were obtained from the Bloomington *Drosophila* Stock Center at Indiana University. The UAS-*TOR^FRB^*, UAS-*S6K^STDETE^*, and UAS-*S6K^KQ^* flies were a gift from Dr. P Kapahi, Buck Institute, Novato, CA.

### Rhodiola Rosea Extract

*Rhodiola rosea* (SHR-5) extract was obtained from the Swedish Herbal Institute. An independent HPLC analysis of this extract was performed by Alkemists Pharmaceuticals (Costa Mesa, CA) as previously described (Schriner et al., 2009a). The formulation used in this study was found to contain 80% *R. rosea* extract and 20% maltodextrin. The whole extract had a 1.7% salidroside content and a 4.5% total rosavin content.

### Feeding and Lifespan Assays

Flies were fed *R. rosea* extract based on the methods described in Jafari *et al*. 2007 [6]. Concentrations of *R. rosea* extract described were dissolved in a yeast solution (4% yeast in 1% acetic acid), and 75 µL of this mixture was overlaid on a banana-molasses food composed of 9% carbohydrate content and a 3.6% yeast content. For the DR studies, dietary yeast contents, in both the banana-molasses food and in the overlaid yeast solution, were as indicated in Figure 1, the concentrations of all other dietary components were unchanged. Flies were maintained at 22±1°C under a 12 h light: 12 h dark cycle for all experiments. For lifespan studies, flies were housed 12 per vial (6 males and 6 females). This density was maintained as long as feasible. Flies were given fresh food every two days and deaths were recorded at these times. Flies were fed *R. rosea* extract at a dose of 25 mg/mL, what we consider the optimal as it confers a maximal lifespan extension in both sexes [7]. Survival analyses were calculated based on the number of deaths recorded and evaluated by the log-rank Mantel-Cox test. Flies were also housed 12 per vial (6 males and 6 females) for all other experiments, independent of the total number needed, and transferred to fresh food every other day.

### Gene Expression Assays

Approximately 400 Flies were fed 25 mg/mL *R. rosea* or an identical control diet for two weeks, and frozen in groups of 50. RNA was extracted with TRIzol reagent (Invitrogen, Carlsbad, CA) according to the manufacturer’s instructions. Samples were treated with DNase (New England Biolabs, Ipswich, MA) at 37°C for 10 min to remove contaminating DNA. DNase was heat inactivated by incubation at 75°C for 10 min in the presence of 5 mM EDTA. RNA was then purified by use of the RNeasy kit (Qiagen, Hilden, Germany). RNA quantity and quality was measured by spectrophotometry. One µg of RNA from each sample was converted to DNA by the iScript cDNA synthesis kit (Bio-Rad, Hercules, CA). Samples were diluted 100-fold. Quantitative PCR was performed on a MiniOpticon real-time PCR system with SYBR green dye (Bio-Rad, Hercules, CA). Relative amplification was calculated by the threshold cycle of each respective gene divided by the threshold cycle of the reference gene, RNA polymerase II. Primer sequences, listed in Table S2, were designed by NCBI/Primer-BLAST. All primers were designed to have a melting temperature of 60°C.

### Pyruvate Kinase Activity

Pyruvate kinase activity was measured by an enzyme-coupled assay through the oxidation of NADH in the presence of phosphoenolpyruvate. One hundred twenty-five µL of a reaction buffer containing 50 mM Imidazole⋅HCl buffer, pH 7.6, 120 mM potassium chloride, 6.2 mM magnesium sulfate, 45 mM ADP, 45 mM phosphoenolpyruvate, 6.6 M NADH, and 1300 units/mL lactate dehydrogenase was added to 25 µL of fly homogenate. Enzyme activity was correlated to the change in absorbance at 340 nm. (Bergmeyer et al., 1974) and normalized to total soluble protein levels. Protein was measured by reaction with Coomassie Brilliant Blue and correlated to a standard curve generated with bovine serum albumin [47].

### Measurement of NAD^+^ and NADH

Approximately 400 Flies were fed 25 mg/mL *R. rosea* with 4% yeast or an identical control diet for two weeks, and frozen in groups of 50. Each group was divided into paired samples of 25 flies and weighed. One of each pair was homogenized in 500 µL of 0.1 N HCl to extract NAD^+^, and the other in 500 µL 0.1 M NaOH to extract NADH. Samples were heated for 5 min at 60°C and centrifuged at 10,000×g for 5 min. To 100 µL of the supernatent, 100 µL of the opposite acid or base solution was added to neutralize the sample, and 10 µL of 1 M Tris, pH 8.0 was added to maintain pH. The NAD^+^ or NADH levels were correlated to the reduction of MTT (570 nm) in the presence of phenazine methosulfate, Ethanol, and alcohol dehydrogenase and compared to a standard curve made with purified NAD^+^ [48]. The final results are presented as pmol NAD^+^ or NADH per mg fly. The ratios of NAD^+^/NADH were calculated by dividing the NAD^+^ content by the respective NADH content within each sample pair of 25 flies.

### Measurement of Protein, Water and Fat Content

Flies were fed for 2 weeks with 25 mg/mL *R. rosea* or a control diet with the indicated concentration of dietary yeast, either 1% or 4%. The flies were then collected with CO_2_ and weighed. For the soluble protein assay, 50 flies per sample were homogenized in 500 µL 100 mM Potassium phosphate buffer, pH 7.4. The samples were centrifuged for 10 min at 10,000×g. Protein in the supernatant was measured by reaction with Coomassie Brilliant Blue and correlated to a standard curve generated with bovine serum albumin [47] and normalized to fly weight. For water content, 10 flies per sample were weighed, dried for 48 h at 70°C, and then weighed again. The difference in weights divided by the initial weight was taken to be the water content.

To determine fat content, the samples used for water measurement were then incubated at RT for 24 h in diethyl ether. The ether was removed and the samples were allowed to dry, and then weighed. Fat content was taken to be the difference in the weights before and after diethyl ether treatment divided by the initial weight (prior to drying at 70°C).

### Starvation and Desiccation Assays

One hundred twenty flies per sex per treatment were fed for two weeks with 25 mg/mL *R. rosea* or control diet. For the desiccation assay, flies were housed in empty vials and deaths were recorded every 2 h. For the starvation assay, flies were housed in vials containing 2% agarose to provide moisture, but no nutritional value. Deaths were recorded every 4 hours. Survival for both assays was determined by log-rank Mantel-Cox test.

### Statistical Analyses

Data were presented as the mean ± sem except for lifespan curves. Statistical analyses were conducted using Prism software (GraphPad, La Jolla, CA). The tests used and sample sizes for each experiment are indicated in the figure captions and in the methods and results sections.

## Supporting Information

Table S1 **Mean and maximum lifespans in** ***Rhodiola rosea*** **-fed and control flies.**
(DOC)

Table S2 **Primer sequences.**
(DOC)
