# Supplementary material for: Extension of Drosophila Lifespan by Rhodiola rosea through a Mechanism Independent from Dietary Restriction
Source: PLoS One. 2013 May 21;8(5):e63886. doi: 10.1371/journal.pone.0063886 (PMC3660385; doi:10.1371/journal.pone.0063886)
Supplement: Table S1 — Mean and maximum lifespans in Rhodiola rosea -fed and control flies. (DOC) [file pone.0063886.s001.doc]

Table S1. Mean and maximum lifespans in *Rhodiola rosea*-fed and control flies.

Experiment Sex Mean Lifespan (Days) Max Lifespan (Days)

Control *R. rosea* Control *R. rosea*

0.1% dietary yeast males 57 71 100 122

0.1% dietary yeast females 75 87 116 132

0.3% dietary yeast males 70 92 110 128

0.3% dietary yeast females 81 94 112 124

1.0% dietary yeast males 69 79 96 110

1.0% dietary yeast females 75 85 96 114

3.0% dietary yeast males 58 72 88 102

3.0% dietary yeast females 59 73 88 110

9.0% dietary yeast males 43 60 76 104

9.0% dietary yeast females 33 45 60 94

*Tub*-GAL4/UAS-*TOR^FRB^* males 51 60 92 104

*Tub*-GAL4/UAS-*S6K^KQ^* males 54 64 100 108

*Tub*-GAL4/UAS-*S6K^STDETE^* males 33 40 56 80

*chico^1^/chico^1^* males 36 41 64 64

*chico^1^/chico^1^* females 47 51 76 72

*Sir2^2A-7-11^/Sir2^2A-7-11^* males 24 30 56 50

*Sir2^2A-7-11^/Sir2^2A-7-11^* females 14 15 30 36
