# Supplementary material for: Extension of Drosophila Lifespan by Rhodiola rosea through a Mechanism Independent from Dietary Restriction
Source: PLoS One. 2013 May 21;8(5):e63886. doi: 10.1371/journal.pone.0063886 (PMC3660385; doi:10.1371/journal.pone.0063886)
Supplement: Table S2 — Primer sequences. (DOC) [file pone.0063886.s002.doc]

Table S2. Primer sequences.

Gene Forward Primer (5’-3’) Reverse Primer (5’-3) Product Size (bp)

RNA pol II AGGGCGGCGAGGACATGGAT CGACGGCTGGTAGTGACCGC 95

dSir2 CGGCCTCGTGCACCAAGTGT CACTGCGGGCACACCGGAAT 91

GAPDH1 GTTGCGGCTGAGGGCGGATT AGTTGATGTTGGCCGGGTCGC 82

Enolase GGCGCCACCAGCTTCACAGA CGGTGGCGTCCAGACCGAAC 97

Pyruvate Kinase TCGCACGAGTACCATGCCGC GCCACGGGGTGTTCGTAGCC 89

dILP2 CGACAGCGATCTGGACGCCC AGGGCACTTCGCAGCGGTTC 93

dILP3 GGCCGCAAACTGCCCGAAAC ACGGGGTCCAAAGTTCTCTTGGT 80

dILP5 AGCAGCAGTTCCAGCAAGGCA GTTTGCGGCCTGGGCGGATA 93

HSP70 ACCAAGGGGTGTGCCCCAGA CTTGGCCTTGCCCGTGCTCA 97

HSP22 TTGGCGGATGGCCGAGGAGA AGCGCCACACTCCAAACGGG 93
